# Myricetin suppresses TGF-β-induced epithelial-to-mesenchymal transition in ovarian cancer

**DOI:** 10.3389/fphar.2023.1288883

**Published:** 2023-11-09

**Authors:** Hui-Wen Yang, Yan Lan, An Li, Han Wu, Zi-Wei Song, Ai-Ling Wan, Yue Wang, Shi-Bao Li, Shuai Ji, Zhong-Cheng Wang, Xin-Yu Wu, Ting Lan

**Affiliations:** ^1^ Xuzhou Key Laboratory of Laboratory Diagnostics, Xuzhou Medical University, Xuzhou, Jiangsu, China; ^2^ School of Medical Technology, Xuzhou Medical University, Xuzhou, Jiangsu, China; ^3^ Department of Laboratory Medicine, Affiliated Hospital of Xuzhou Medical University, Xuzhou, Jiangsu, China; ^4^ School of Pharmacology, Xuzhou Medical University, Xuzhou, Jiangsu, China; ^5^ Department of Pathophysiology, School of Basic Medical Sciences, Xuzhou Medical University, Xuzhou, Jiangsu, China; ^6^ Department of Laboratory Medicine, Affiliated Xuzhou Maternity and Child Health Care Hospital of Xuzhou Medical University, Xuzhou, China

**Keywords:** myricetin, epithelial-to-mesenchymal transition, TGF-β, ovarian cancer, PI3K/AKT, TGF-β/Smad

## Abstract

**Background:** Ovarian cancer (OC) is the second most common gynecological malignancy and has a high mortality rate. The current chemotherapeutic drugs have the disadvantages of drug resistance and side effects. Myricetin, a kind of natural compound, has the advantages of easy extraction, low price, and fewer side effects. Multiple studies have demonstrated the anti-cancer properties of myricetin. However, its impact on OC is still unknown and needs further investigation. Therefore, this study aimed to elucidate the mechanism by which myricetin suppresses transforming growth factor-β (TGF-β) -induced epithelial-to-mesenchymal transition (EMT) in OC through *in vivo* and *in vitro* experiments.

**Methods:** *In vitro* experiments were conducted to evaluate the effects of myricetin on cell proliferation and apoptosis using CCK8 assay, plate clonal formation assay, and flow cytometry. Western blot was employed to evaluate the expression levels of caspase-3, PARP, and the MAPK/ERK and PI3K/AKT signaling pathways. Wound healing, transwell, western blot and immunofluorescence assay were used to detect TGF-β-induced cell migration, invasion, EMT and the levels of Smad3, MAPK/ERK, PI3K/AKT signaling pathways. Additionally, a mouse xenograft model was established to verify the effects of myricetin on OC *in vivo*.

**Results:** Myricetin inhibited OC proliferation through MAPK/ERK and PI3K/AKT signaling pathways. Flow cytometry and western blot analyses demonstrated that myricetin promoted apoptosis by increasing the expression of cleaved-PARP and cleaved-caspase-3 and the ratio of Bax/Bcl-2 in OC. Furthermore, myricetin suppressed the TGF-β-induced migration and invasion by transwell and wound healing assays. Mechanistically, western blot indicated that myricetin reversed TGF-β-induced metastasis through Smad3, MAPK/ERK and PI3K/AKT signaling pathway. *In vivo*, myricetin significantly repressed OC progression and liver and lung metastasis.

**Conclusion:** Myricetin exhibited inhibitory effects on OC progression and metastasis both *in vivo* and *in vitro*. And it also reversed TGF-β-induced EMT through the classical and non-classical Smad signaling pathways.

## 1 Introduction

OC is one of the most lethal gynecological malignancies. It is the eighth most common cancer for women and the second leading cause of gynecological cancer death reported by the International Agency for Research on Cancer in 2020 in 185 countries [Sung et al., 2021]. Due to symptoms being generally insidious, approximately two-thirds of OC are diagnosed at Stage III or Stage IV, meaning that patients usually have already developed metastases [Torre et al., 2018; Berek et al., 2021]. Recently, a flavonoid compound called myricetin has been reported to have anti-cancer effects in breast, colon, and hepatocellular carcinomas [Ci et al., 2018; Shiomi et al., 2013; Ma et al., 2019]. Besides, many studies indicated that myricetin has anti-inflammatory, anti-oxidative, anti-platelet aggregation, and cardioprotective effects [ Semwal et al., 2016; Agraharam et al., 2022]. Tavsan et al. demonstrated that myricetin, functioning as an anti-oxidant, induced apoptosis in human OC cells by reducing ROS levels effectively [Li et al., 2022; Tavsan and Kayali, 2019]. Myricetin also leads to apoptosis in OC cells through DNA double-strand breaks (DSBs) and endoplasmic reticulum (ER) stress [Xu et al., 2016]. Moreover, Tavsan et al. found that myricetin inhibited proliferation by arresting the cell cycle in OC cells [Tavsan and Kayali, 2019]. To date, few studies have been able to elaborate on the specific mechanism by which myricetin affects OC.

EMT is one of the main reasons for metastasis and progression in OC. During the EMT process, tumor cells undergo a series of changes, including loss of cell junctions and polarity, cytoskeleton reorganization, altered cell shape, increased motility and protrusion, and enhanced ability to degrade extracellular matrix (ECM) proteins. The above alteration is a sophisticated biological process resulting from the induction of transcription factors and the activation of respective signal pathways [Lamouille et al., 2014]. A hallmark of EMT is the downregulation of E-cadherin and upregulation of N-cadherin. Through this process, epithelial cells lose adhesion junctions and acquire mesenchymal stem cell-like properties [Cavallaro and Christofori, 2004; Vergara et al., 2010]. Various factors and signaling pathways, including microRNAs, growth factors, MMPs, and TGF-β, contribute to the induction of EMT. Notably, it has been reported that high levels of TGF-β within ascites are related to EMT, leading to OC metastasis [Sicard et al., 2021]. The TGF-β superfamily includes TGF-β, activins, bone morphogenetic proteins (BMPs), and other secreted cytokines. Evolving evidence elucidated that TGF-β triggered EMT in carcinoma cells through Smad and non-Smad signaling pathways. Upon binding to its receptor heteromer, TGF-β sequentially activates Smad2/3 and Smad4 in turn. The trimeric Smad complex translocates into the nucleus and induces the transcription of key transcription factors. Then, the Smad complex cooperates with the transcription regulators, such as Snail, to regulate target gene expression. Furthermore, TGF-β can activate other signaling molecules such as MAPK, PI3K/AKT, PAK2, and RhoA in a cell type-dependent manner [Xu et al., 2016; Gonzalez and Medici, 2014]. In this study, we aimed to investigate whether myricetin suppressed migration and invasion in OC cells by reversing EMT. We also identified the specific signaling pathways involved. Our results suggested that myricetin reserved EMT of OC cells induced by TGF-β via Smad-mediated and non-Smad signaling *in vitro* and *in vivo*.

## 2 Materials and methods

### 2.1 Reagents and antibodies

Myricetin (Aladdin, Shanghai, China) has the chemical formula C15H10O8, a relative molecular mass of 318.24, and a purity of >98%. TGF-β was purchased from MedChemexpress (MCE, Shanghai, China). Anti-rabbit IgG, anti-mouse IgG, and antibodies against GAPDH, ERK, p-ERK, AKT, p-AKT, Bax, Bcl-2, PARP, E-cadherin, Snail, Vimentin were purchased from Proteintech (Proteintech, Chicago, United States). Antibodies against cleaved-PARP, cleaved-caspase-3, were purchased from Cell Signal Technology (Danvers, MA, United States). Antibodies against Smad3 and p-Smad3 were purchased from Affinity Biosciences (Affinity Biosciences, OH, United States). The antibody against N-cadherin was purchased from Servicebio (Servicebio, Wuhan, China).

### 2.2 Cell culture

Human OC cell lines HO8910 and A2780 and the human normal epithelial cell line IOSE80 were obtained from the American Type Culture Collection (ATCC). IOSE80 cell lines were cultured in Roswell Park Memorial Institute-1640 (RPMI- 1640) medium (Kaiji, Jiangsu, China) with 10% fetal bovine serum (FBS) and 1% penicillin-streptomycin (P/S, Invitrogen).

OC cells were cultured in Dulbecco’s modified Eagle medium (DMEM) (Kaiji, Jiangsu, China) containing 10% fetal bovine serum (Gibco, Grand Island, NY, United States). Cell culture was performed at 37°C with 5% CO2 in the air. When cell fusion reached 70%–80%, EMT was induced by treating 10 ng/mL TGF-β1 with the DMEM. Meanwhile, EMT was administered at appropriate concentrations of myricetin for 24 h.

### 2.3 CCK8

A2780, HO8910, and IOSE80 cells at the logarithmic growth stage were selected, and the cell concentration was adjusted to 2×10^4^/mL. The cells were inoculated into 96-well plates with 100 μL per well. After the cells were attached to the wall, different concentrations of myricetin and CCK-8 reagent (Kaiji, Jiangsu, China) were added to each well and cultured in an incubator for 24 h. The mixture was incubated for 1h, and an enzyme marker detected the absorbance at 450 nm.

### 2.4 Colony formation

A2780, HO8910, and IOSE80 cells at the logarithmic growth stage were selected, the cell concentration was adjusted to 1×10^3^/mL, and the cells were inoculated into the six-well plate, each well was 1 mL. After the cells were attached to the wall, different concentrations of myricetin were added to each well, and the cells were cultured in the incubator for 10–15 days. The culture was terminated when the number of single-cell colonies in the six-well plate was greater than or equal to 50. Next, the medium was discarded, and the sample was washed with PBS 3 times and then fixed with 4% paraformaldehyde for 15 min. Afterward, it was washed with PBS three more times and stained with crystal violet for 15 min. The sample was then rinsed with deionized water, air-dried at room temperature, photographed and analyzed.

### 2.5 Western blot

After the cells were treated with TGF-β or myricetin of different concentrations, the cell precipitates of each group were collected. An appropriate amount of RIPA lysate was added and fully lysed on ice for 30 min. The mixture was violently shaken for 1 min every 10 min, centrifuged at 12 000rpm for 30 min at 4°C, and the supernatant was collected. The protein concentration was determined by BCA method. 20μg protein was isolated by SDS-PAGE electrophoresis. Then, proteins were transferred to PVDF membrane and incubated overnight with primary antibody diluent at 4°C after being sealed with 5% skim milk for 1 h at room temperature. The next day, after washing the membrane, the second antibody diluent labeled by HRP was added and incubated at room temperature for 1 h. The membrane reacted with the chemiluminescence reagent after washing it, and protein expression was visualized using the chemiluminescence imaging system. Image Lab software was used to process and analyze its gray value.

### 2.6 Cell apoptosis

Cells treated with myricetin at different concentrations were digested with trypsin digestion solution without EDTA and treated according to Annexin V-EGFP/PI double staining apoptosis detection Kit (Kaiji, Jiangsu, China). Flow cytometry was used for detection and Flow Jo analysis.

### 2.7 Wound healing

A straight line across the hole was marked with a marker on the back of the six-well plate, and cells of the logarithmic growth stage were selected. The medium was discarded, and cells were washed with PBS thrice. After incubating with serum-free medium for 12 h, cells in each hole were drawn in three straight lines using a 10 μL sterile gun tip. TGF-β and different concentrations of myricetin were added into each hole, and the six-well plate was placed in the incubator for continued culture. Photos were taken at the same position at 0, 24, and 48 h respectively.

### 2.8 Transwell

The invasion experiment was coated with the basement membrane, Matrigel matrix glue was diluted with the serum-free medium at 1:5, and 100 μL dilution glue was added into the upper chamber and placed in the incubator for 4–6 h. Logarithmic growth cells were taken, and the concentration of cells was adjusted to 2×10^5^/mL by serum-free medium. 200μL cell suspension was absorbed and added to the upper chamber, and the cell suspension was directly absorbed and added to the upper chamber of the migration experiment. 600 μL complete medium, TGF-β, and different concentrations of myricetin were added to the lower chamber and placed in the incubator. The migration experiment was 24 h and the invasion experiment was 48 h. Fixed with 4% paraformaldehyde for 15 min, washed with PBS three times, stained with crystal violet for 15 min, rinsed with deionized water, and gently wiped the unpenetrated cells in the upper chamber with a cotton swab. The picture is under a 200-power microscope.

### 2.9 Immunofluorescence staining

Cells of the logarithmic growth stage were selected and the cell concentration was adjusted to 2×10^4^/mL. The cells were inoculated into 24-well plates, with each well containing 300 μL. After the cells were attached to the wall, the medium was discarded and the serum-free medium was added for 12 h. Fixed in 4% paraformaldehyde for 15min, permeated with 0.1%Triton X-100 for 10min, closed with 5% BSA for 2 h, incubated with anti-E-cadherin and anti-N-cadherin overnight at 4°C, added corresponding fluorescent secondary antibody, 37°C for 1h, DAPI dye for 10min. Photographs were taken with a fluorescence microscope.

### 2.10 Tumor xenograft

The 6-week-old female nude mice were purchased from the Animal Center of Xuzhou Medical University. A2780 cells (5×10^6^) were resuscitated with 200 µL 1% matrix (Corning, USA) and injected subcutaneously into the back of nude mice. After the tumor was formed, the nude mice were given myricetin gavage every other day. The tumor was weighed, and HE and immunohistochemistry staining were performed.

### 2.11 Immunohistochemistry

Tumor tissue from nude mice was dewaxed, rehydrated and incubated in 3% H2O2 for 15 min at room temperature and protected from light to block peroxidase. Antigen repair was performed in citric acid antigen repair buffer (PH = 6) followed by blocking with 10% BSA and incubated with antibodies against Ki67, MMP9 or EGFR (Affinity, Shanghai, China) overnight at 4°C, followed by secondary antibody for 50 min at room temperature. Drops of chromogenic solution were added, hematoxylin was restrained and the slices were dehydrated and blocked. The images were obtained with Olympus microscope and image analysis was performed.

### 2.12 H&E staining

The liver and lung tissues were fixed with 4% paraformaldehyde for at least 12 h. After dehydration, the tissues were embedded in paraffin, cut into 5 μm, and then routinely stained with hematoxylin and eosin (H&E). All slides were photographed using a light microscope (Olympus, Japan, Tokyo).

### 2.13 Ethical statement

The animal study was reviewed and approved by the Ethics Committee of the Experimental Animal Research Center of Xuzhou Medical University (202207S015).

### 2.14 Statistical analysis

Each experiment was performed at least three times. Data were shown as mean ± standard deviation (SD) and analyzed by GraphPad Prism software (version 8.0.2). The statistical comparison between groups was conducted via Student’s t-tests or one-way analysis of variance (ANOVA). *p < 0.05; **p < 0.01; ***p < 0.001; ****p < 0.0001 were considered statistically significant.

## 3 Results

### 3.1 Effect of myricetin on the proliferation of OC cells A2780 and HO8910 as well as normal ovarian epithelium cells

**FIGURE 1:**
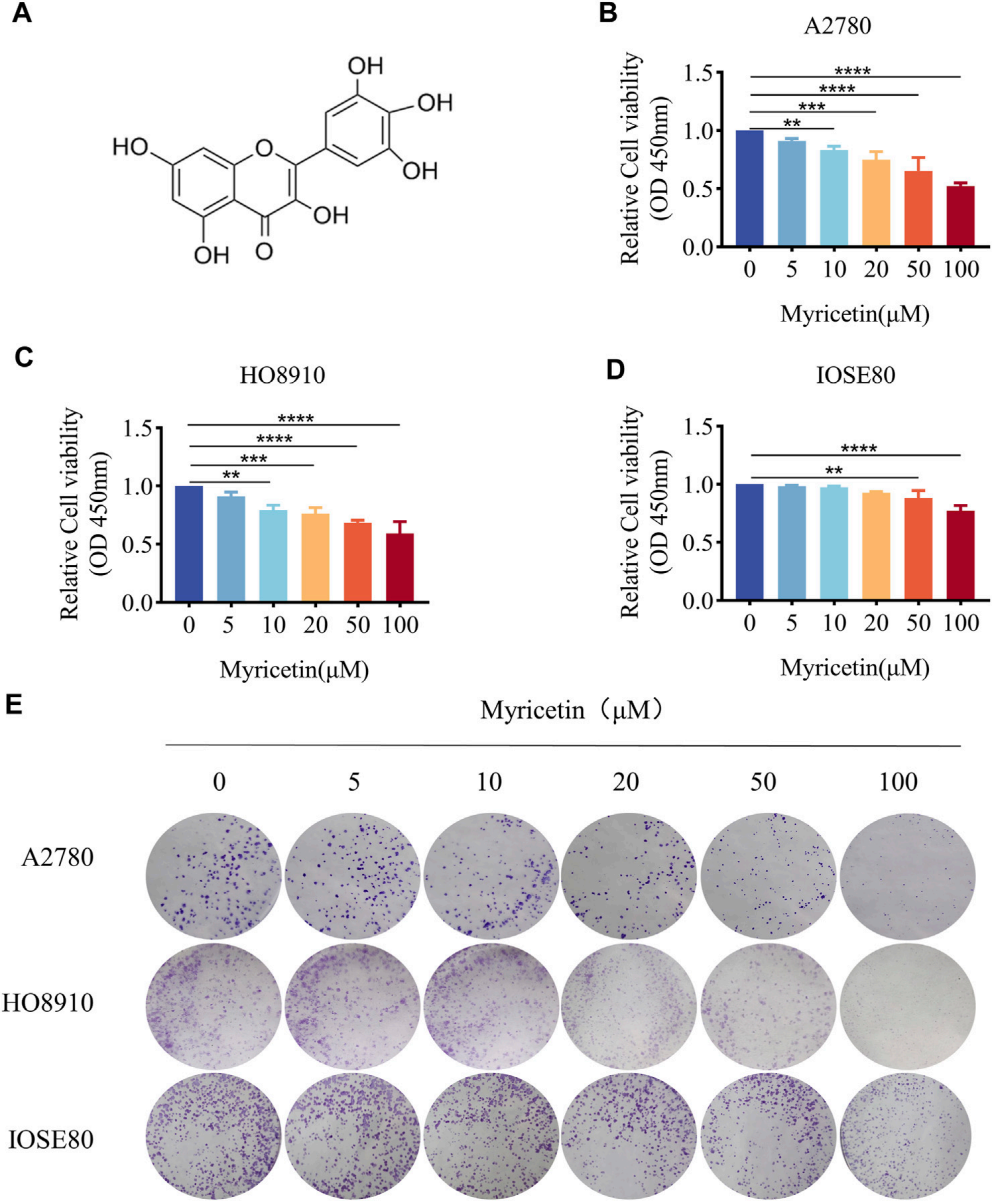
Myricetin inhibited ovarian cancer proliferation. **(A)** Chemical Structure of myricetin. **(B)** A2780 **(C)** HO8910 **(D)** and IOSE80 Cell proliferation was analyzed by CCK8 assays. **(E)** The ability of cells to proliferate was assayed with the colony formation assay. *p < 0.05; **p < 0.01; ***p < 0.001; ****p < 0.0001 were considered statistically significant, n = 3.

CCK-8 assay was employed to assess the effect of myricetin on OC and normal ovarian epithelium cell lines. All 3 cell lines were treated with five different concentrations of myricetin (5μM, 10μM, 20μM, 50μM, 100 μM) for 48 h. OC cells A2780 and HO8910 exhibited significant inhibition of cell viability when treated with concentrations above 10 μM myricetin (Figures 1B,C), with respective IC50 values of 117.1μM and 202.1 μM (supplied Figure 1). In contrast, there was minimal effect on the proliferation of normal ovarian epithelial cells IOSE80 compared to A2780 or HO8910 cells, and no significant alteration in cell proliferation at a concentration below 50 μM (Figure 1D). Additionally, the number and area of clone formation in A2780 and HO8910 cells were markedly suppressed by myricetin concentrations above 10μM, while normal ovarian epithelial cells IOSE80 showed little effect on clone formation (Figure 1E). Therefore, we selected the concentrations of 5, 10, and 20 μM myricetin for further experiments. In summary, both the CCK-8 and plate cloning assays demonstrated that myricetin inhibited the proliferation capacity of OC cells in a concentration-dependent manner.

### 3.2 Myricetin inhibits the phosphorylation level in the MAPK/ERK and PI3K/AKT signaling pathways

**FIGURE 2:**
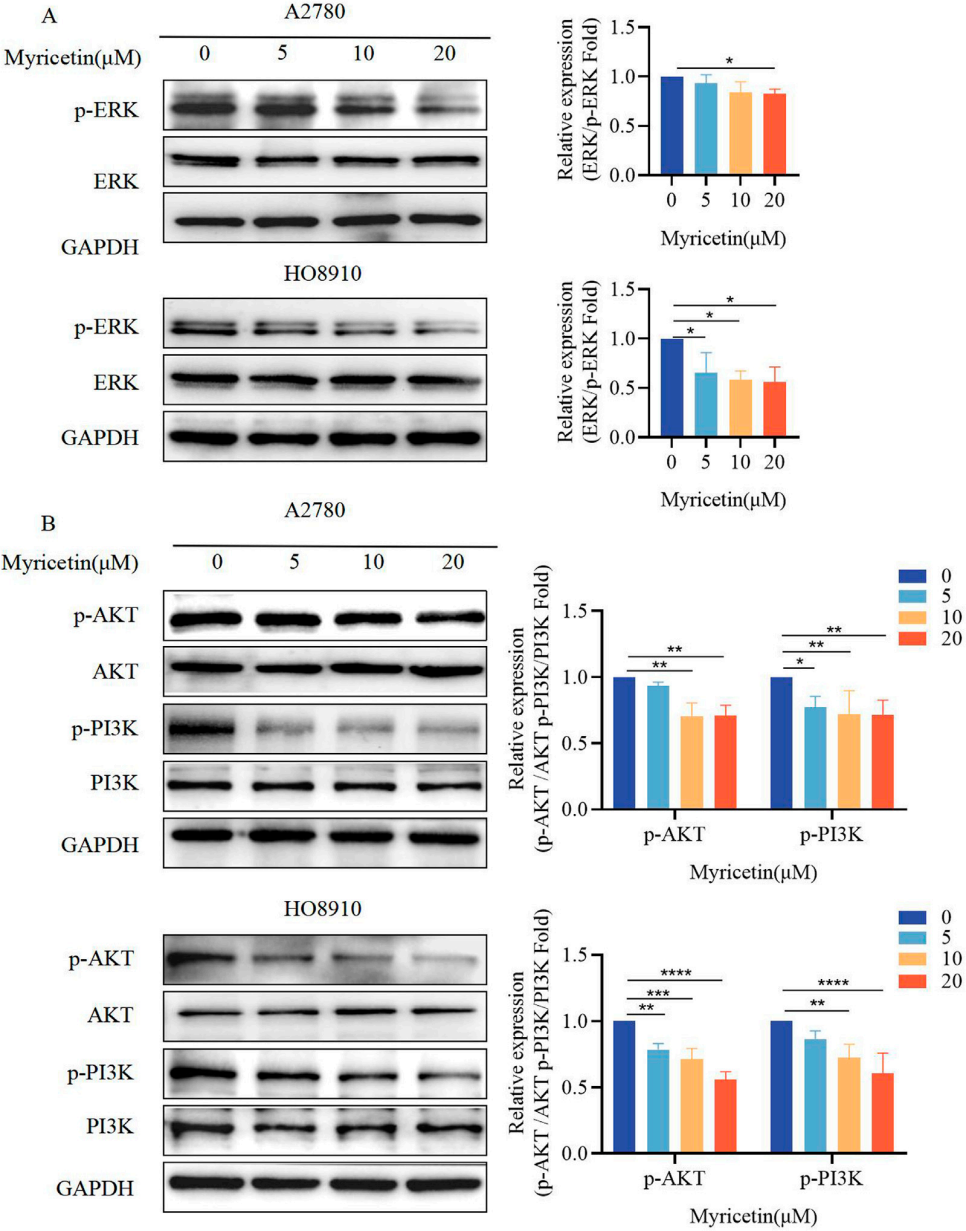
Myricetin inhibited the phosphorylation level of MAPK/ERK and PI3K/AKT signaling pathways. Analysis of the level of **(A)** p-ERK and ERK and **(B)** p-PI3K, PI3K, p-AKT, and AKT in OC cells (A2780 and HO8910) by western blotting. *p < 0.05; **p < 0.01; ***p < 0.001; ****p < 0.0001 were considered statistically significant, n = 3.

Previous research has demonstrated that myricetin exhibits importance in colon cancer tumorigenesis and progression, suggesting that its mechanisms may involve the activation of the ERK/MAPK and PI3K/AKT signaling pathways [Zhu et al., 2020]. Western blot analysis was conducted to determine whether myricetin exhibits similar effects on MARK/ERK and PI3K/AKT in OC. The results indicated that myricetin downregulated phosphorylated ERK and PI3K/AKT in A2780 and HO8910 cells (Figures 2A,B). Furthermore, we observed that myricetin inhibited the activation of ERK/MAPK and PI3K/AKT signaling pathways in a concentration-dependent manner.

### 3.3 Myricetin induces apoptosis of OC cell lines A2780 and HO8910

**FIGURE 3:**
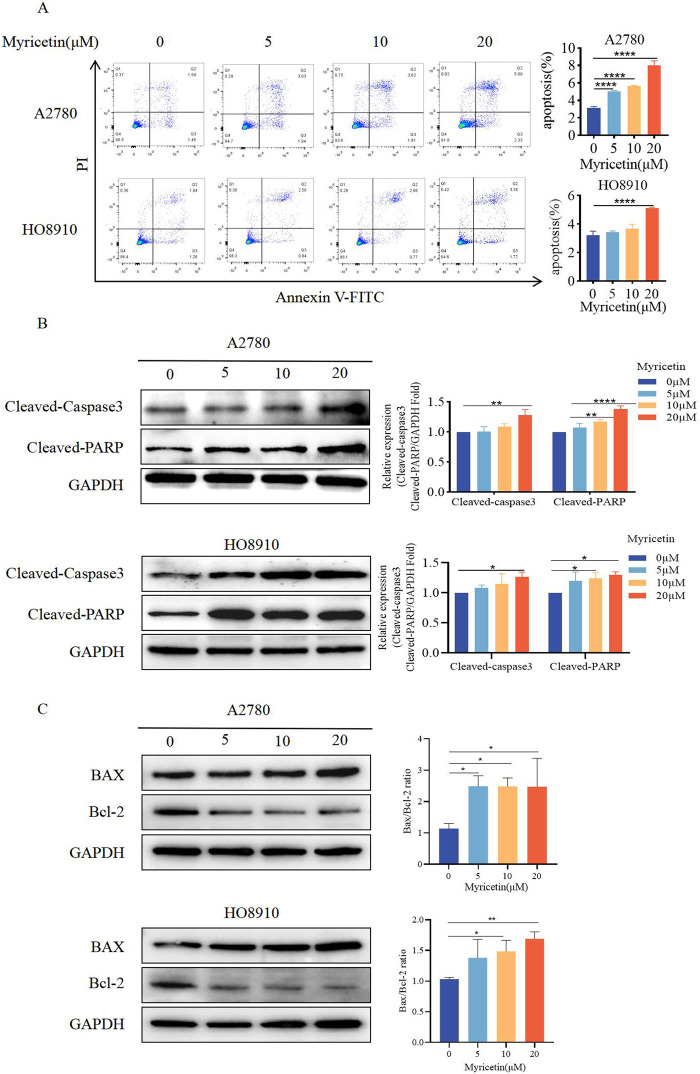
Myricetin promoted apoptosis of OC cell lines. **(A)** The apoptosis levels of A2780 and HO8910 cells were determined by Flow cytometry after myricetin treatment. **(B)** Analysis of the expression level of cleaved-caspase-3 and cleaved-PARP in OC cells (A2780 and HO8910) by western blotting. **(C)** Analysis of the expression level of Bax and Bcl-2 in OC cells (A2780 and HO8910) by western blotting.*p < 0.05; **p < 0.01; ***p < 0.001; ****p < 0.0001 were considered statistically significant, n = 3.

Cells were analyzed by Annexin V-FITC/PI co-staining flow cytometry (Figure 3A) to determine whether myricetin suppressed the viability of A2780 and HO8910 cells by inducing apoptosis. The results showed that myricetin reduced the percentage of viable cells while increasing the percentage of apoptotic cells in a dose-dependent manner. Western blot analysis further validated these results (Figure 3B). Caspase-3 and its substrate PARP play a crucial role in cell apoptosis. Following myricetin treatment, the levels of cleaved-caspase-3 and cleaved-PARP were found to be higher than those in the control group. The Bcl-2/Bcl-XL (anti-apoptotic) and Bax and Bak (pro-apoptotic), part of the Bcl-2 family proteins, regulate apoptosis, and the relative ratio of these proteins can either inhibit or promote apoptosis (Kale et al., 2018). Previous reports have also indicated that myricetin induces apoptosis by increasing the Bax/Bcl-2 ratio in breast cancer and lymphoma (Han et al., 2022; Song et al., 2021). Furthermore, our results indicated that myricetin elevated Bax levels while decreasing Bcl-2 levels, resulting in an upregulation of the Bax/Bcl-2 ratio (Figure 3C). These findings collectively demonstrate that myricetin promotes apoptosis in OC by enhancing the expression of cleaved-PARP and cleaved-caspase-3, as well as increasing the Bax/Bcl-2 ratio.

### 3.4 Myricetin inhibits OC growth and metastasis *in vivo*

**FIGURE 4:**
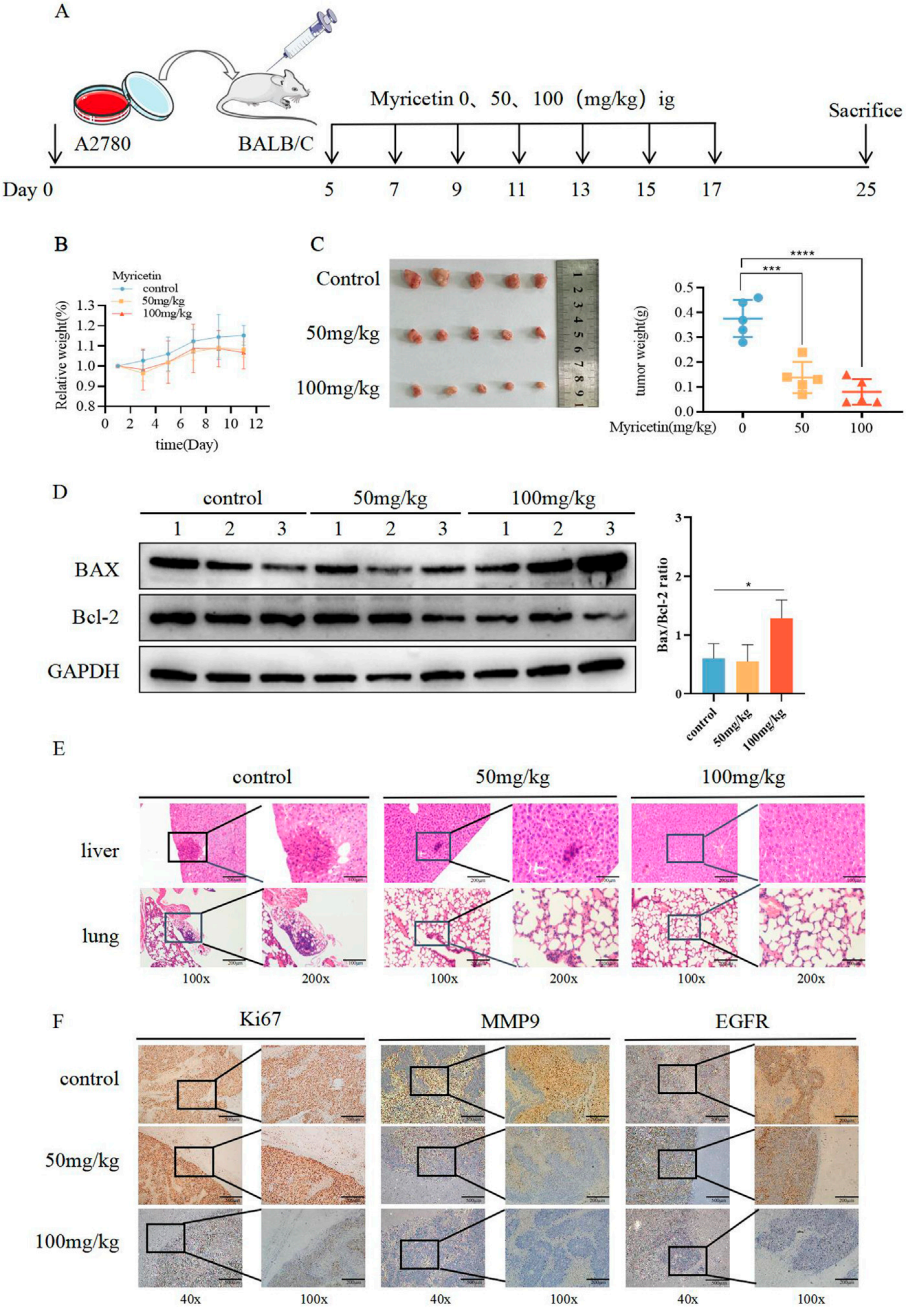
Suppressive influence of myricetin in the growth of OC cells *in vivo*. **(A)** Construction of subcutaneous heterograft tumor model in nude mice. **(B, C)** The change of tumor weight and size in model mice. **(D)** Analysis of the expression level of Bax and Bcl-2 in tumor by western blotting. **(E)** Analysis of liver and lung metastasis in model mice by HE staining. **(F)** Analysis of the level of EGFR, Ki-67, and MMP-9 in tumor tissues of nude mice by immunohistochemistry. *p < 0.05; **p < 0.01; ***p < 0.001; ****p < 0.0001 were considered statistically significant, n = 5.

To investigate the role of myricetin *in vivo*, we established the subcutaneous xenograft model of the A2780 cells in nude mice. Once tumors formed, myricetin was administered every other day via gavage for a total of seven doses (Figure 4A). The results demonstrated a noticeable reduction in tumor volume after myricetin administration, especially at the 100 mg/kg dosage, while the mice showed no adverse effects on body weight (Figures 4B,C), indicating that myricetin inhibited tumor growth without causing harm to the nude mice. Furthermore, we investigated whether myricetin promotes apoptosis *in vivo* by comparing Bax and Bcl-2 protein expression levels among the control group, 50 mg/kg group and 100 mg/kg group. The results showed that following intragastric administration of myricetin, there was an increase in Bax levels, a decrease in Bcl-2 levels, and corresponding elevation in the Bax/Bcl-2 ratio (Figure 4D). Additionally, the results of H&E staining showed that myricetin markedly inhibited the liver and lung metastasis in 50 mg/kg and 100 mg/kg groups (Figure 4E). Moreover, immunohistochemical staining of the tumor tissue was performed, compared with the control group, the expressions of Ki-67, MMP9, and EGFR in the myricetin treatment group were significantly decreased (Figure 4F). These results collectively suggest that myricetin inhibits the growth and metastasis of OC cells *in vivo*.

### 3.5 Myricetin inhibits TGF-β-induced metastasis and EMT progression in OC cells

**FIGURE 5:**
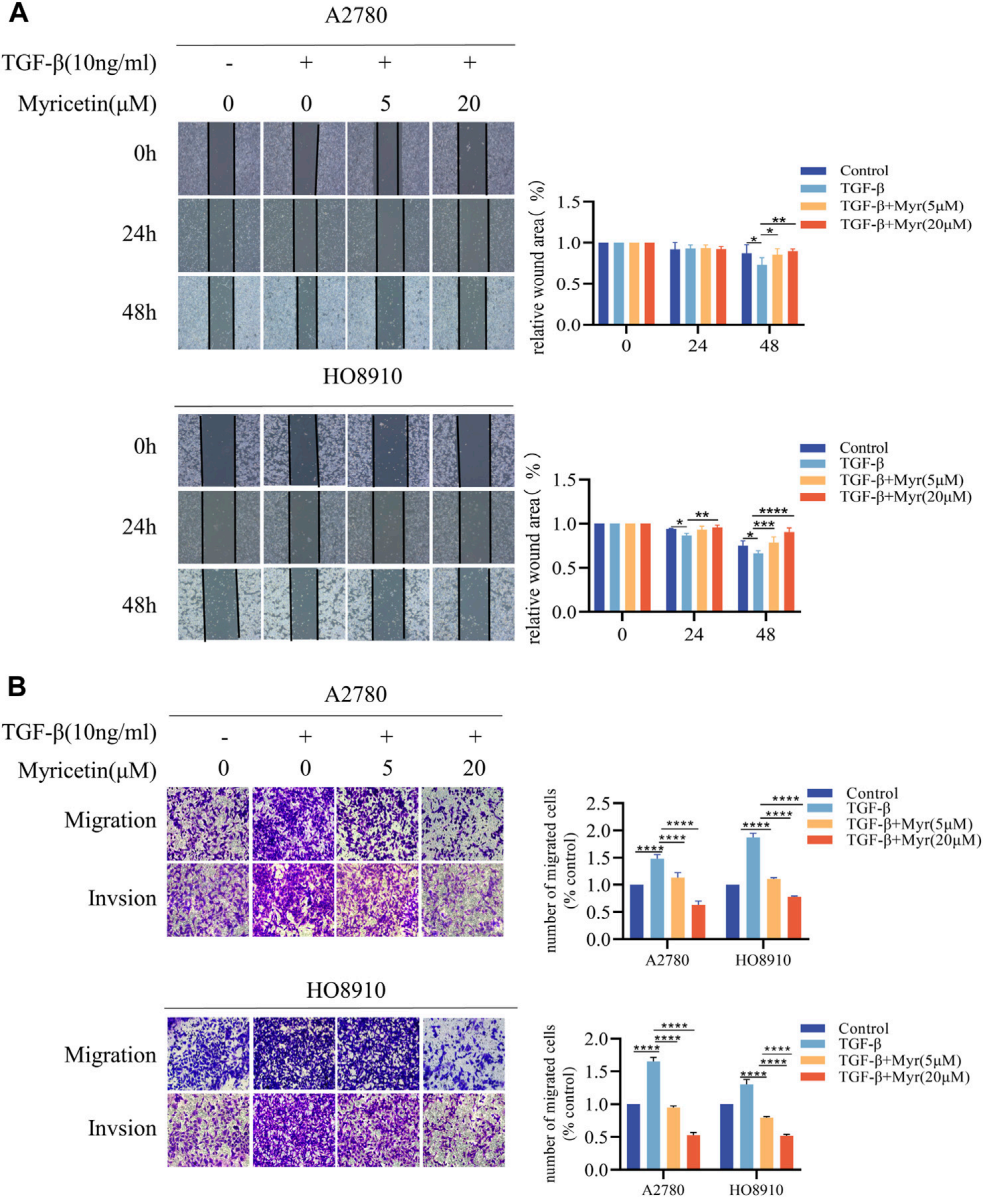
Myricetin inhibits TGF-β-induced migration and invasion in ovarian cancer cells. **(A)** Representative results of wound healing assays. The bar chart represents the migration distance. Error bars represent the means ± SD of three independent experiments (5 fields/experiment).**(B)** Changes in A2780 and HO8910 cells migration and invasion after treatment with TGF-β alone or in combination with myricetin by transwell assay.*p < 0.05; **p < 0.01; ***p < 0.001; ****p < 0.0001 were considered statistically significant, n = 3.

**FIGURE 6:**
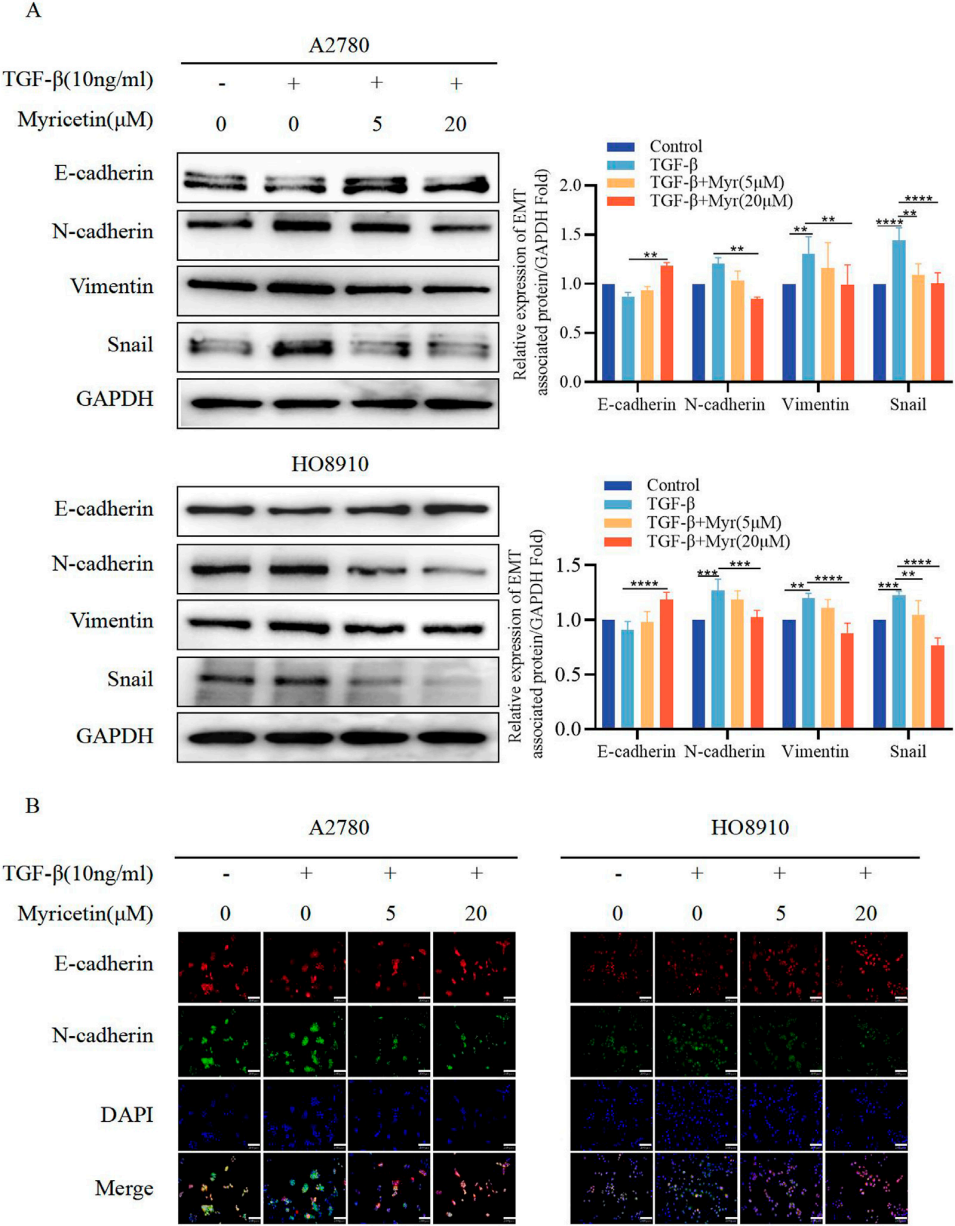
Myricetin reverses TGF-β-induced EMT to inhibit the metastasis and invasion of OC cells. **(A)** Analysis of the expression level of E-cadherin, N-cadherin, Vimentin, and Snail in OC cells by western blot. **(B)** The expression levels of E-cadherin and N-cadherin in A2780 and HO8910 cells were detected by immunofluorescence assay.*p < 0.05; **p < 0.01; ***p < 0.001; ****p < 0.0001 were considered statistically significant, n = 3.

To further clarify the inhibitory effect of myricetin on TGF-β-induced OC metastasis, we performed wound healing assays. We added 10 ng/mL TGF-β or co-cultured TGF-β and myricetin (5 μM or 20 μM) to A2780 and HO8910 to observe the cell migration at 24h and 48 h. As shown in Figure 5A, TGF-β induced a significant reduction in cell spacing, whereas adding different concentrations of myricetin led to increased cell spacing. Subsequently, we confirmed these findings through transwell migration and invasion assay (Figure 5B). To verify whether myricetin restrains metastasis of OC cells by reversing EMT, we investigated EMT-related molecules. Western blot and immunofluorescence staining results showed that compared with TGF-β-untreated cells, the expression of E-cadherin was diminished, whereas the expression of the mesenchymal cell markers (N-cadherin, Vimentin, and Snail) was increased in TGF-β-treated cells (Figures 6A,B). Moreover, TGF-β-induced E-to-N-cadherin switching was suppressed by myricetin. These results revealed that myricetin inhibited OC metastasis by reversing EMT induced by TGF-β.

### 3.6 Myricetin inhibits TGF-β-induced EMT in OC cells through the classical smad signaling pathway

**FIGURE 7:**
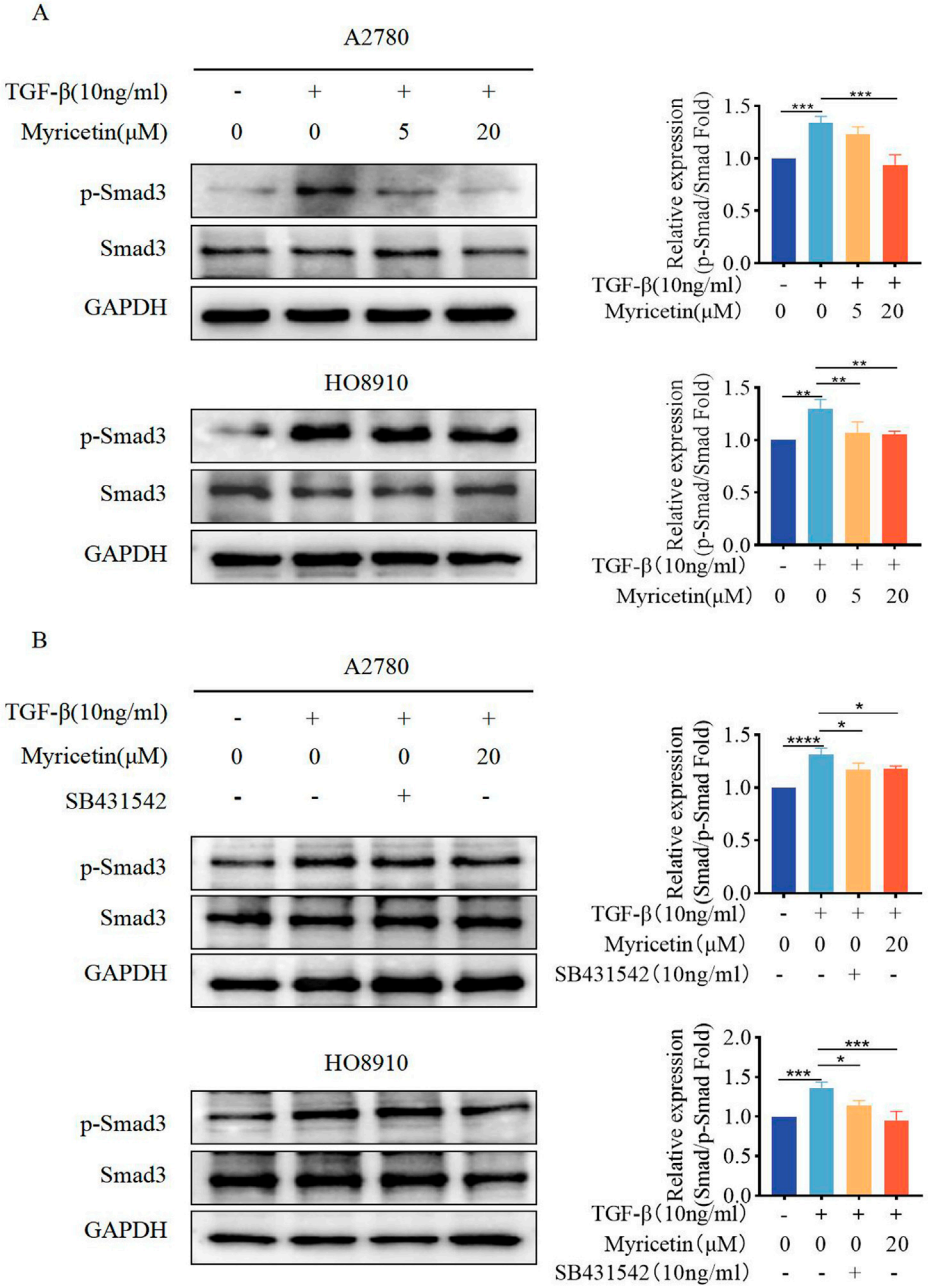
Myricetin inhibits TGF-β-induced EMT in OC cells through the classical Smad signaling pathway. **(A)** Analysis of the expression level of phosphorylated Smad3 and Smad3 in OC cells. **(B)** Analysis of the phosphorylated Smad3 and Smad3 in OC cells after different combination treatments of TGF-β, myricetin, or SB431542(10 ng/mL). *p < 0.05; **p < 0.01; ***p < 0.001; ****p < 0.0001 were considered statistically significant, n = 3.

As TGF-β-induced EMT was associated with the Smad signaling pathway, we investigated whether myricetin modulated this classical TGF-β pathway. Both cell lines were treated with TGF-β (10 ng/mL) and varying concentrations of myricetin (5μM, 20 μM). Our results showed that TGF-β upregulated p-Smad3, but the level of p-Smad3 was decreased after administering myricetin (Figure 7A). Next, we combined TGF-β with either TGF-β/Smad inhibitor SB431542 or 20 μM myricetin. As shown in Figure 7B, myricetin exhibited similar effects to the TGF-β/Smad inhibitor SB431542, resulting in the downregulation of p-Smad3 induced by TGF-β in both cell lines. In conclusion, myricetin inhibited TGF-β-induced EMT in OC cells through the Smad signaling pathway.

### 3.7 Myricetin inhibits TGF-β-induced EMT in OC cells through ERK/MAPK and PI3K/AKT signaling pathways

**FIGURE 8:**
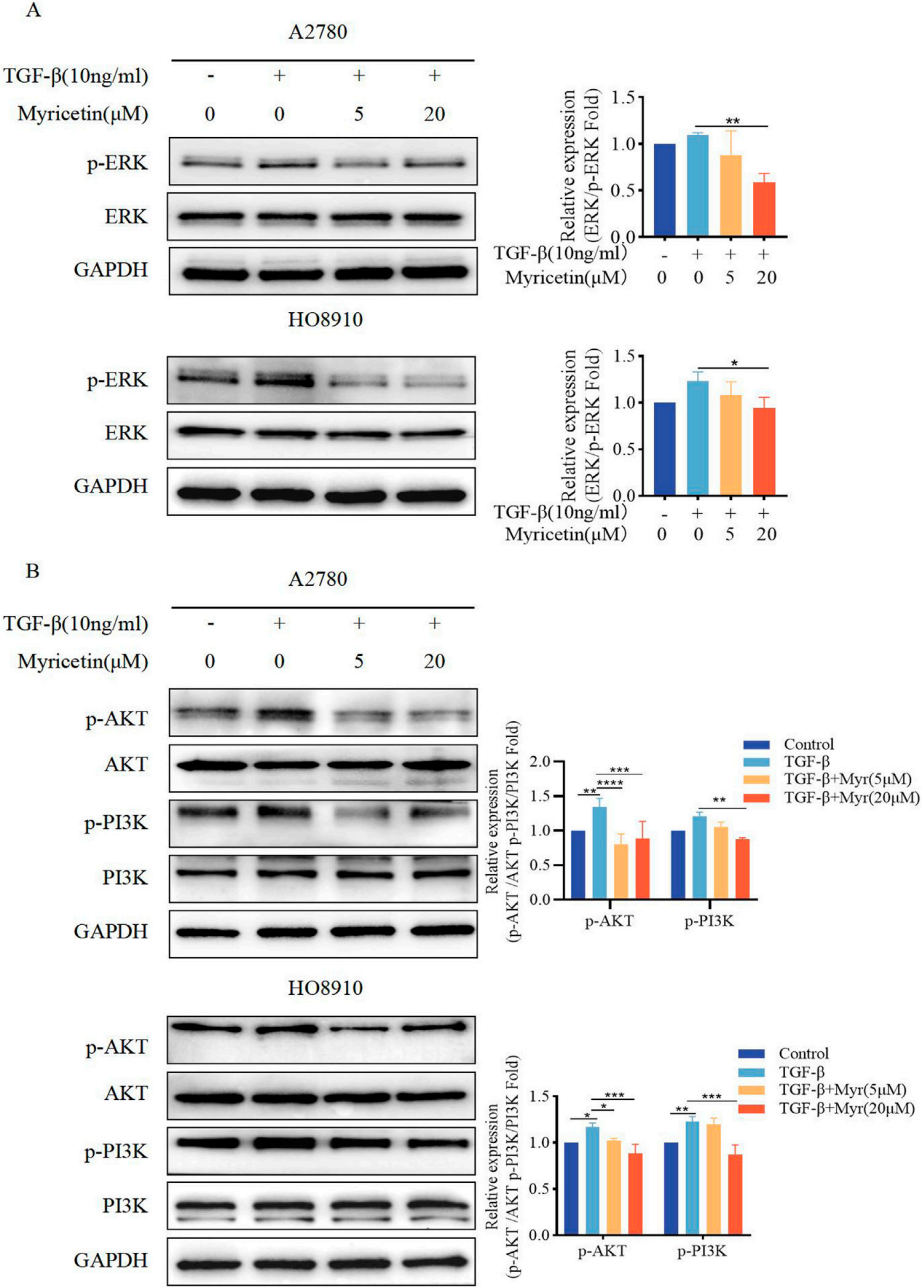
Myricetin inhibits TGF-β-induced EMT in OC cells through ERK/MAPK and PI3K/AKT signaling pathways. **(A)** Analysis of the expression level of phosphorylated ERK and ERK in OC cells. **(B)** Analysis of the expression level of phosphorylated PI3K, PI3K, phosphorylated AKT, and AKT in OC cells. Results are presented as mean ± SD (n = 5). *p < 0.05; **p < 0.01; ***p < 0.001; ****p < 0.0001 were considered statistically significant, n = 3.

To assess whether myricetin suppressed EMT through the non-Smad signaling pathways, phosphorylation of ERK, AKT, and PI3K was measured under the same condition. We found that A2780 and HO8910 cells significantly increased in p-AKT and p-PI3K after TGF-β treatment. Notably, myricetin, added at either 5 μM or 20 μM concentration, demonstrated inhibitory activity compared to the TGF-β-treated group (Figures 8A, B). Thus, myricetin inhibited TGF-β-mediated EMT through ERK/MAPK and PI3K/AKT signaling pathways.

## 4 Discussion

Currently, paclitaxel is the primary first-line treatment for OC. However, issues like drug resistance, relapse and metastasis remain significant challenges. Therefore, there is an urgent need to explore new natural treatments for OC. Our research has uncovered that myricetin inhibits OC proliferation, promotes apoptosis, and inhibits metastasis both *in vivo* and *in vitro*. Myricetin achieves this by reversing TGF-β-induced EMT, acting through both classical Smad and non-classical pathways. In conclusion, our study identifies myricetin as a promising natural treatment option for OC.

Recent studies have suggested edible flavonoids as potential anti-cancer candidates. Myricetin, as a flavonol drug, was found in a wide variety of natural sources. Cytotoxicity assays revealed that myricetin had minimal toxic effects on ovarian epithelial cells but exhibited a stronger inhibitory effect on OC cells. This indicates that myricetin selectively targets cancer cells while sparing normal cells, establishing it as a promising natural treatment for OC. Several studies have demonstrated that myricetin has anti-tumor effects in various cancers, including gastric, breast, colorectal, and ovarian cancers. (Feng et al., 2015; Han et al., 2022; Jiang et al., 2019; Kim et al., 2014). It induced apoptosis and cell cycle arrest in gastric cancer cells via Ribosomal S6 kinase 2 and Mad1 (Feng et al., 2015). Myricetin downregulated matrix metalloproteinase-2/9 activity and ST6GALNAC5 expression, inhibiting migration and invasion in breast cancer [Ci et al., 2018]. Additionally, myricetin induced apoptosis in colon cancer cells through the Bax/Bcl-2-dependent pathway (Kim et al., 2014). In OC, myricetin has been found to suppress cancer cell proliferation and migration [Zheng et al., 2017]. Recently, Qi Li et al. corroborated these findings, demonstrating that myricetin reduced migratory and aggressive capacity in OC [Li et al., 2022]. These previous reports are consistent with our experimental results. Myricetin inhibited OC proliferation through PI3K/AKT and MAPK/ERK pathways. It is well known that PI3K/AKT and MAPK/ERK pathways participate in various cellular processes, including cell growth, proliferation, and apoptosis. Numerous studies have revealed that activation of these pathways is associated with oncogenesis and tumor proliferation [Nunnery and Mayer, 2020]. In lung cancer cells, the phosphorylation levels of PI3K/AKT/mTOR are linked to tumor cell migration [Ko et al., 2018]. Furthermore, evidence suggests that curcumol hinders breast cancer growth by regulating the PI3K/AKT pathway [Zhou et al., 2023]. In our study, we employed Flow cytometry and observed that myricetin induced apoptosis in OC cells. S. H. Han et al. reported that myricetin induced apoptosis via the MAPK pathway in breast cancer [Han et al., 2022]. Additionally, some researchers have noted that myricetin triggers apoptosis by increasing the Bax/Bcl-2 ratio and releasing AIF from mitochondria into the cytosol [Kim et al., 2014]. In our study, it is noteworthy that myricetin elevated Bax levels decreased Bcl-2 levels, and upregulated the Bax/Bcl-2 ratio. Additionally, myricetin promoted apoptosis through the PARP/caspase-3 pathway. During apoptosis, initiator caspases (e.g., Caspases-8 and -9) are initially activated, followed by effector caspases (e.g., caspases −3 and-7), which are cleaved and activated by the promoter. Subsequently, effector caspases cleaved structural proteins, leading to cell death [Yadav et al., 2021]. The DNA repair enzyme poly (ADP-ribose) polymerase (PARP) is a classical substrate of caspase-3 and can detect DNA strand breaks. caspase-3 and PARP, especially cleaved-caspase-3 and cleaved-PARP, play key roles in the regulation of apoptosis [Wang et al., 2018]. Our results indicated that myricetin promoted apoptosis in OC cells through the caspase cascade. Subsequently, we further confirmed that myricetin inhibited tumor growth and promoted apoptosis in subcutaneous xenograft models. Moreover, it was observed that myricetin significantly reduced OC metastasis in the liver and intestine. Therefore, we aimed to elucidate the specific mechanism of myricetin inhibition of OC metastasis.

Clinically, the majority of ovarian cancers exhibit metastasis upon detection, with ascites facilitating the transfer of OC to the liver and intestines. As OC cells detach into ascites, TGF-β induces epithelial mesenchymal transformation (EMT), causing the loss of epithelial characteristics and the acquisition of mesenchymal traits, enhancing migration, invasion, and apoptosis resistance. Myricetin has demonstrated the ability to reverse EMT in liver cancer, transforming MHCC97H cells from EMT to MET in concentration-dependent and inhibiting hepatocellular carcinoma migration and invasion [Lee et al., 2019]. Our study similarly revealed that myricetin reversed TGF-β-induced EMT process in OC. Previous studies suggests that both canonical and non-canonical signaling pathways induce EMT in cancer cells. Canonical signaling involves receptor activation, leading to C-terminal phosphorylation of transcription factors known as Smad, regulating gene expression [Semwal et al., 2016]. In non-canonical signaling, TGF-β activates downstream signaling pathways, like p38 MAPK, ERK1/2, and PI3K/AKT pathways [Semwal et al., 2016; Agraharam et al., 2022; Varela-Rodríguez et al., 2020]. These pathways collectively activate transcription factors, including Snail1, Slug, ZEB1/2, and Twist1/2, ultimately downregulate epithelial cell markers such as E-cadherin and promoting the expression of mesenchymal cell markers such as N-cadherin and vimentin [Ci et al., 2018; Shiomi et al., 2013; Varela-Rodríguez et al., 2020]. Various natural compounds have been documented to inhibit TGF-β1-induced EMT and metastasis through the non-canonical signaling pathway and canonical pathways. Won et al. reported that polysaccharides from persimmon leaf inhibit TGF-β-induced EMT by targeting canonical Smad2/3 and non-canonical ERK/p38 signaling pathways in A549 cells [Lim et al., 2020]. Isoviolanthin extracted from Dendrobium officinale leaves targets the TGF-β/Smad and PI3K/AKT/mTOR pathways to suppress TGF-β1-induced EMT in hepatocellular carcinoma (HCC) cells. Osthole mediated the EMT by down-regulating Snail and cell-invasive capability, suppressing the TGF-β/AKT/MAPK pathway [Xing et al., 2018]. We used a TGF-β/Smad inhibitor to confirm that myricetin inhibited TGF-β-induced phosphorylation of Smad3 in OC cells. Our results also manifested that myricetin significantly attenuated the phosphorylation of MAPK/ERK and the PI3K/AKT signaling pathway induced by TGF-β.

## 5 Conclusion

In summary, our study revealed that myricetin impeded the growth of OC *in vivo*. *In vitro* experiments, myricetin restrained OC cell proliferation by down-regulating PI3K/AKT and MAPK/ERK signaling pathways. Additionally, it induced OC cell apoptosis via the PARP/caspase-3 and Bax/Bcl-2 pathway. Besides, myricetin effectively reverses the TGF-β-induced EMT progression through classical and non-classical Smad signaling pathways, inhibiting OC cell metastasis. This study suggests that myricetin is a potential natural treatment for OC. However, this study has certain limitations. For instance, we merely list the possible mechanisms of myricetin inhibiting OC without conducting further research. Therefore, in future studies, we will intensify our exploration of the molecular mechanisms through which myricetin inhibits TGF-β-induced OC metastasis, encompassing both Smad and non-Smad signaling pathways.

## Data Availability

The raw data supporting the conclusion of this article will be made available by the authors, without undue reservation.
