# Corrigendum: Myricetin suppresses TGF-β-induced epithelial-to-mesenchymal transition in ovarian cancer

**DOI:** 10.3389/fphar.2024.1447095

**Published:** 2024-11-14

**Authors:** Hui-Wen Yang, Yan Lan, An Li, Han Wu, Zi-Wei Song, Ai-Ling Wan, Yue Wang, Shi-Bao Li, Shuai Ji, Zhong-Cheng Wang, Xin-Yu Wu, Ting Lan

**Affiliations:** ^1^ Xuzhou Key Laboratory of Laboratory Diagnostics, Xuzhou Medical University, Xuzhou, Jiangsu, China; ^2^ School of Medical Technology, Xuzhou Medical University, Xuzhou, Jiangsu, China; ^3^ Department of Laboratory Medicine, Affiliated Hospital of Xuzhou Medical University, Xuzhou, Jiangsu, China; ^4^ School of Pharmacology, Xuzhou Medical University, Xuzhou, Jiangsu, China; ^5^ Department of Pathophysiology, School of Basic Medical Sciences, Xuzhou Medical University, Xuzhou, Jiangsu, China; ^6^ Department of Laboratory Medicine, Affiliated Xuzhou Maternity and Child Health Care Hospital of Xuzhou Medical University, Xuzhou, China

**Keywords:** myricetin, epithelial-to-mesenchymal transition, TGF-β, ovarian cancer, PI3K/AKT, TGF-β/Smad

In the published article, there were errors in “Figures 2, 3, 6–8” as published. Certain images were mixed between groups, resulting in the unintentional misplacement of the representative images in Figures 2B, 3A, 6A, 7A, B, 8A. The corrected Figures 2, 3, 6, 7, 8 and their captions appear below.

**FIGURE 2:**
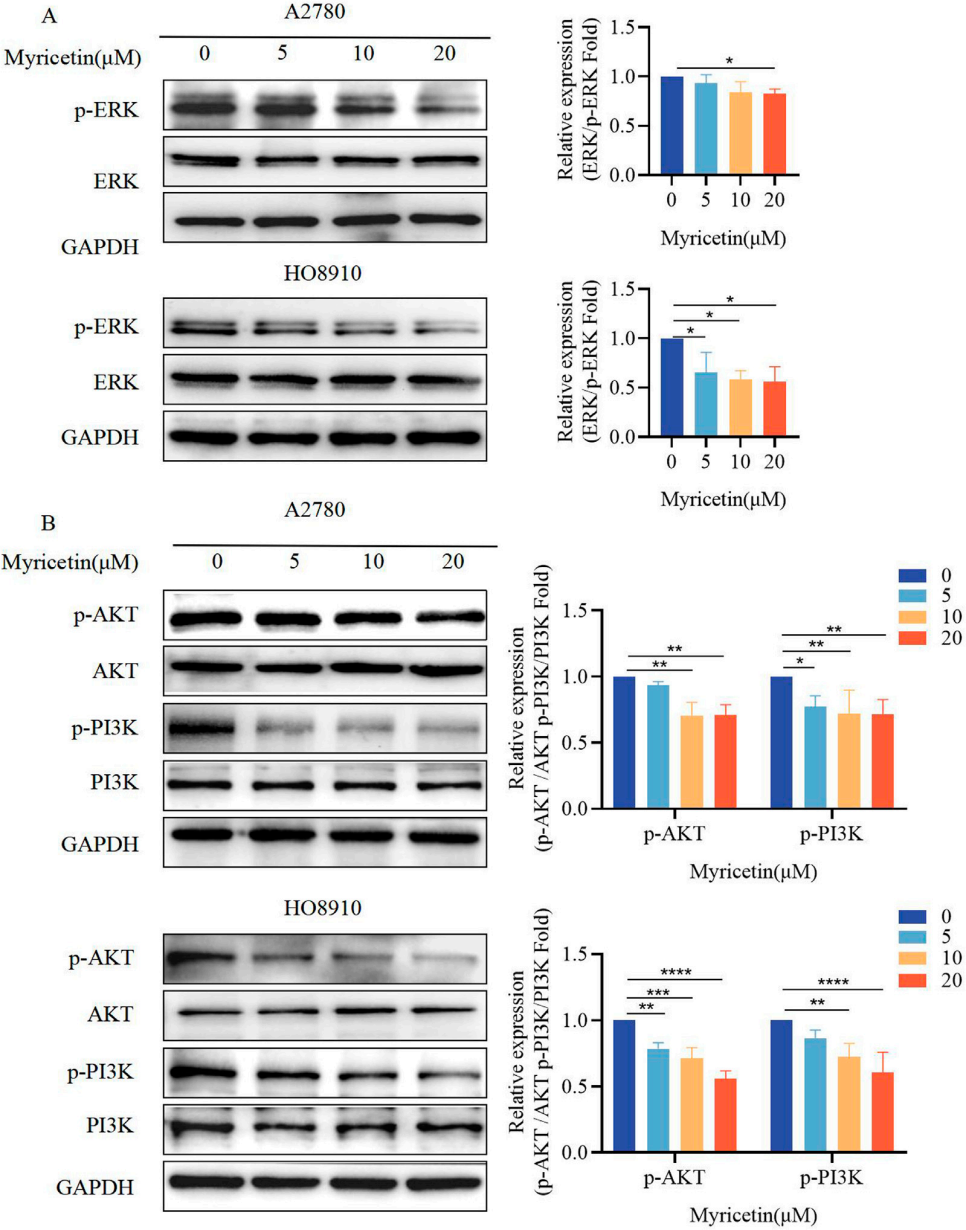
Myricetin inhibited the phosphorylation level of MAPK/ERK and PI3K/AKT signaling pathways. Analysis of the level of **(A)** p-ERK and ERK and **(B)** p-PI3K, PI3K, p-AKT, and AKT in OC cells (A2780 and HO8910) by western blotting. *p < 0.05; **p < 0.01; ***p < 0.001; ****p < 0.0001 were considered statistically significant, n = 3.

**FIGURE 3:**
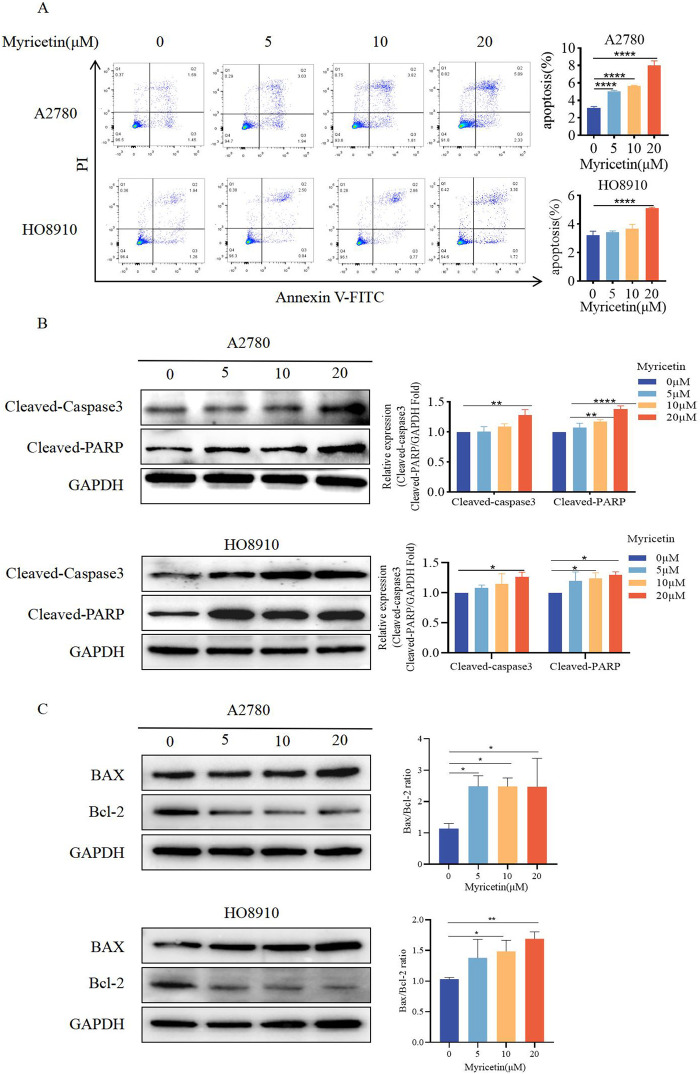
Myricetin promoted apoptosis of OC cell lines. **(A)** The apoptosis levels of A2780 and HO8910 cells were determined by Flow cytometry after myricetin treatment. **(B)** Analysis of the expression level of cleaved-caspase-3 and cleaved-PARP in OC cells (A2780 and HO8910) by western blotting. **(C)** Analysis of the expression level of Bax and Bcl-2 in OC cells (A2780 and HO8910) by western blotting.*p < 0.05; **p < 0.01; ***p < 0.001; ****p < 0.0001 were considered statistically significant, n = 3.

**FIGURE 6:**
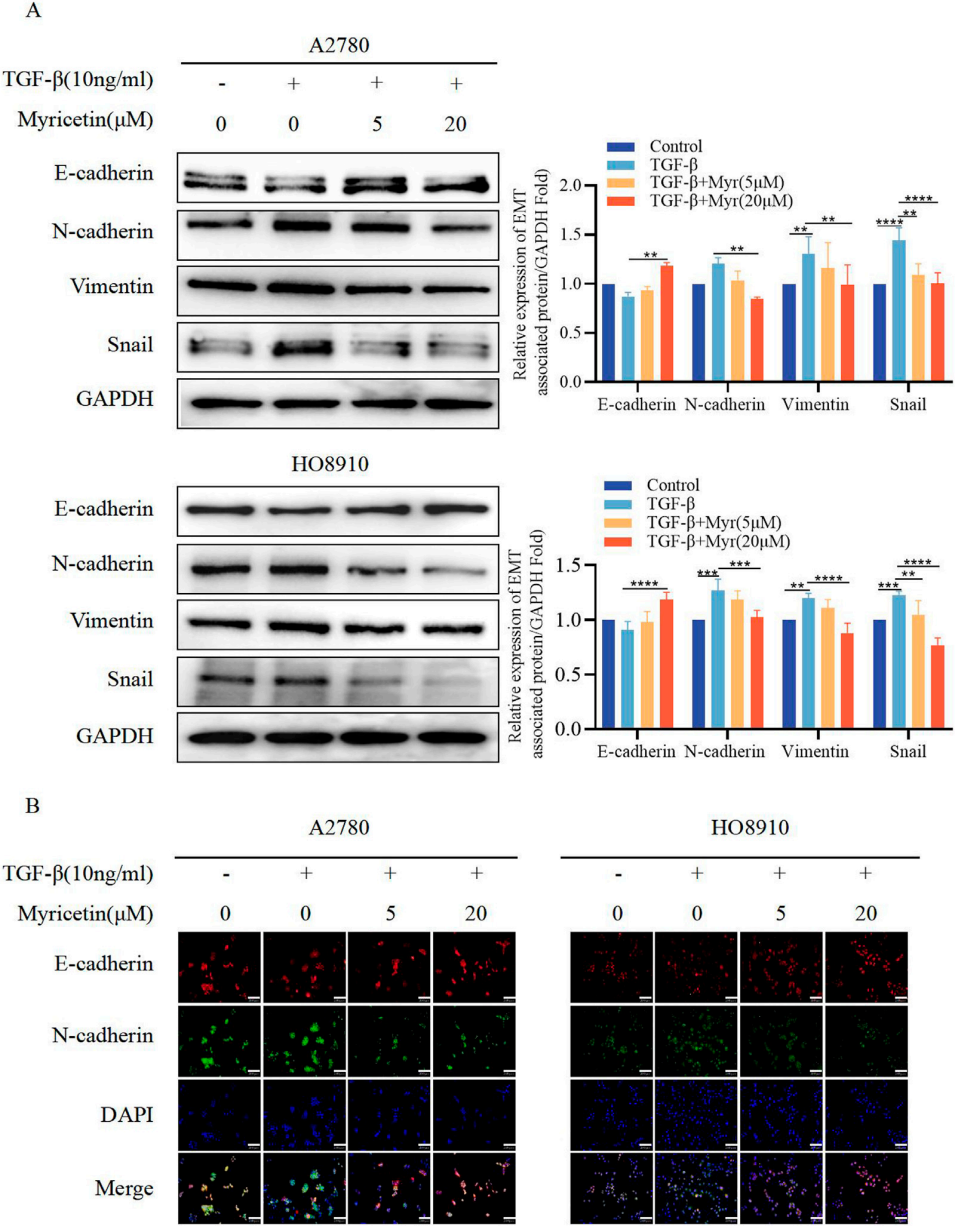
Myricetin reverses TGF-β-induced EMT to inhibit the metastasis and invasion of OC cells. **(A)** Analysis of the expression level of E-cadherin, N-cadherin, Vimentin, and Snail in OC cells by western blot. **(B)** The expression levels of E-cadherin and N-cadherin in A2780 and HO8910 cells were detected by immunofluorescence assay.*p < 0.05; **p < 0.01; ***p < 0.001; ****p < 0.0001 were considered statistically significant, n = 3.

**FIGURE 7:**
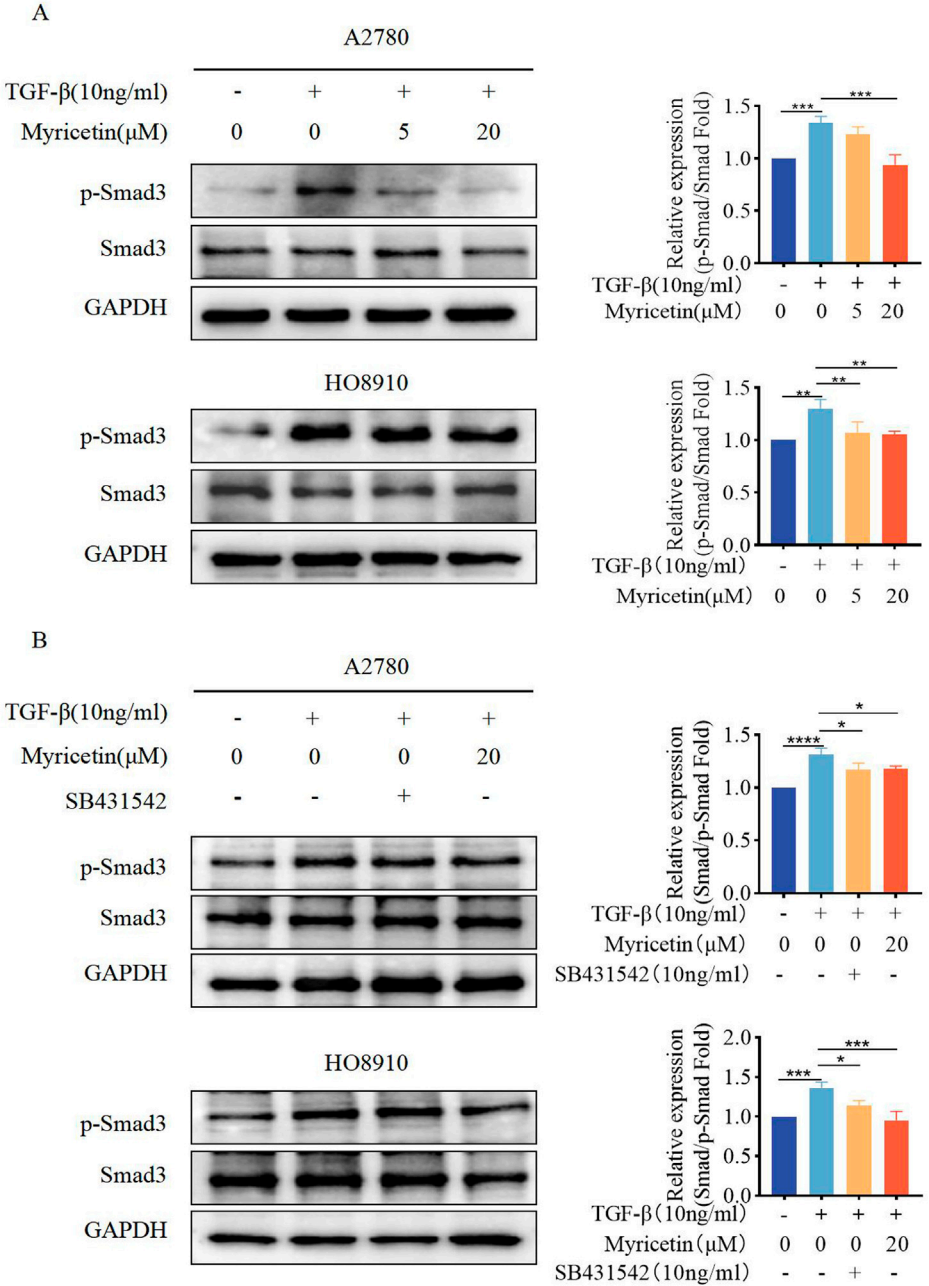
Myricetin inhibits TGF-β-induced EMT in OC cells through the classical Smad signaling pathway. **(A)** Analysis of the expression level of phosphorylated Smad3 and Smad3 in OC cells. **(B)** Analysis of the phosphorylated Smad3 and Smad3 in OC cells after different combination treatments of TGF-β, myricetin, or SB431542(10 ng/mL). *p < 0.05; **p < 0.01; ***p < 0.001; ****p < 0.0001 were considered statistically significant, n = 3.

**FIGURE 8:**
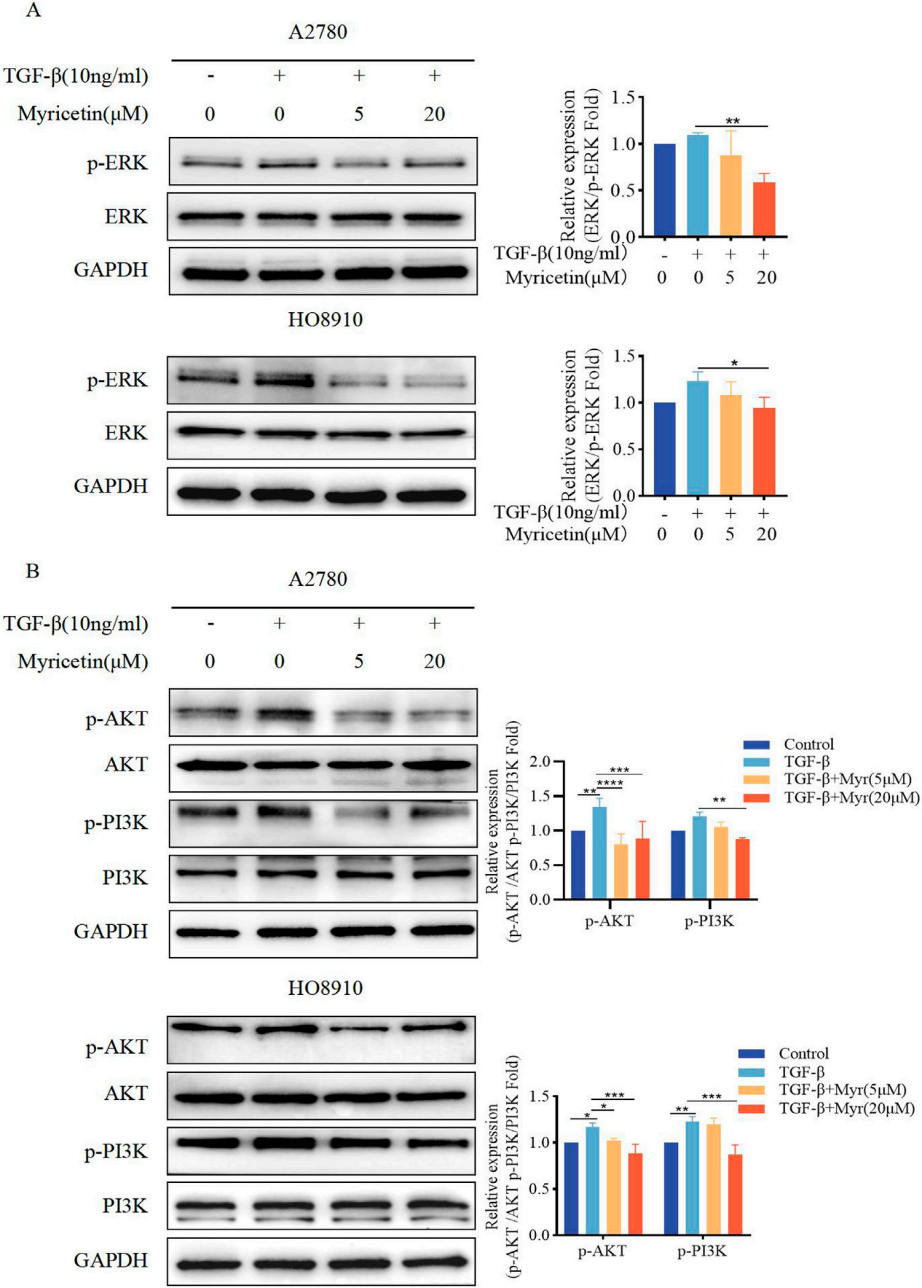
Myricetin inhibits TGF-β-induced EMT in OC cells through ERK/MAPK and PI3K/AKT signaling pathways. **(A)** Analysis of the expression level of phosphorylated ERK and ERK in OC cells. **(B)** Analysis of the expression level of phosphorylated PI3K, PI3K, phosphorylated AKT, and AKT in OC cells. Results are presented as mean ± SD (n = 5). *p < 0.05; **p < 0.01; ***p < 0.001; ****p < 0.0001 were considered statistically significant, n = 3.

In the published article, there was an error in Figure 4 as published. The tumors in the control group exceeded the usual size in Figures 4A–C. The corrected Figure 4 and its caption appear below.

**FIGURE 4:**
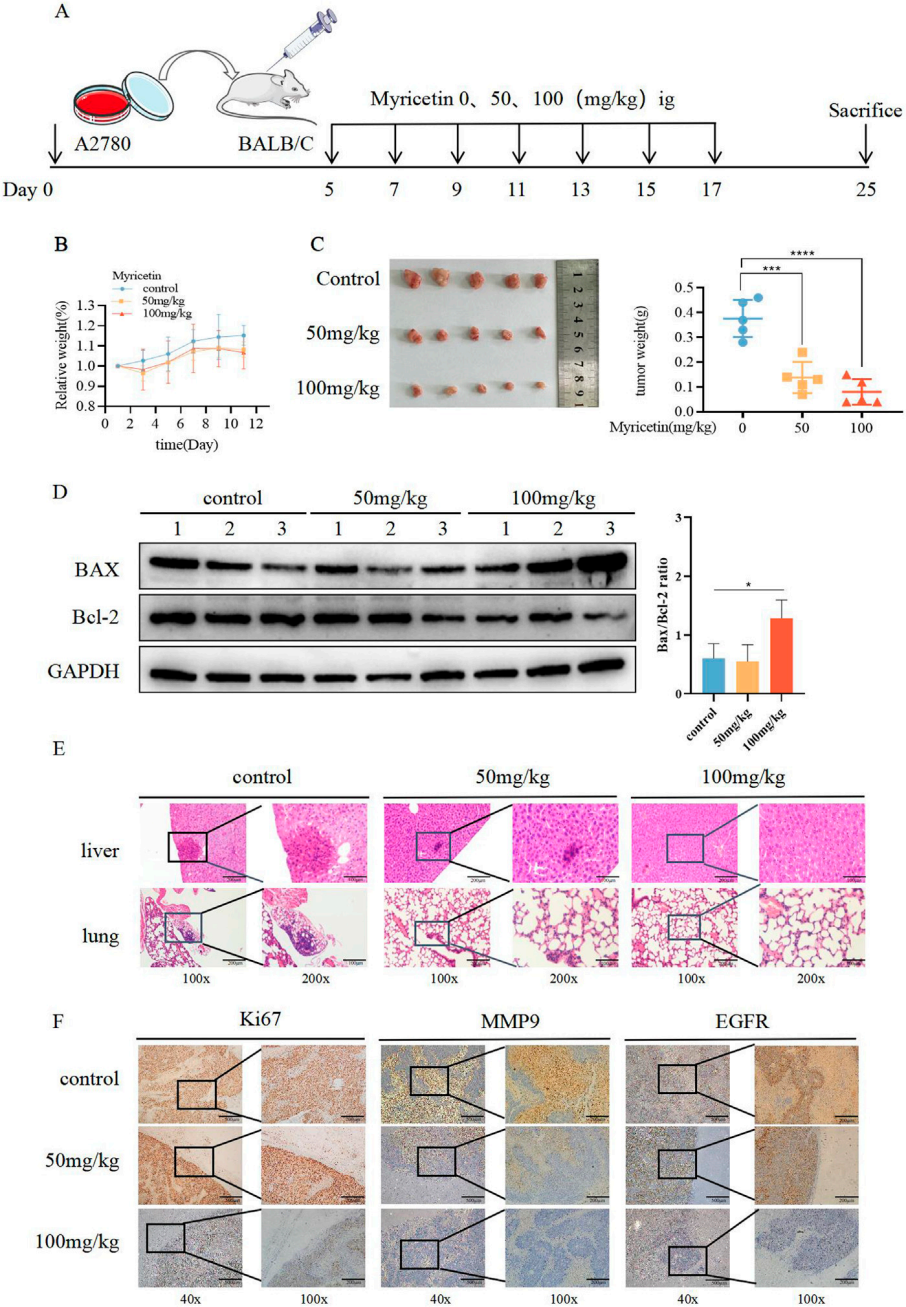
Suppressive influence of myricetin in the growth of OC cells *in vivo*. **(A)** Construction of subcutaneous heterograft tumor model in nude mice. **(B, C)** The change of tumor weight and size in model mice. **(D)** Analysis of the expression level of Bax and Bcl-2 in tumor by western blotting. **(E)** Analysis of liver and lung metastasis in model mice by HE staining. **(F)** Analysis of the level of EGFR, Ki-67, and MMP-9 in tumor tissues of nude mice by immunohistochemistry. *p < 0.05; **p < 0.01; ***p < 0.001; ****p < 0.0001 were considered statistically significant, n = 5.

The authors apologize for these errors and state that these do not change the scientific conclusions of the article in any way. The original article has been updated.

