# Supplementary figures and images for: Myricetin suppresses TGF-β-induced epithelial-to-mesenchymal transition in ovarian cancer
Source: Front Pharmacol. 2023 Nov 9;14:1288883. doi: 10.3389/fphar.2023.1288883 (PMC10665490; doi:10.3389/fphar.2023.1288883)

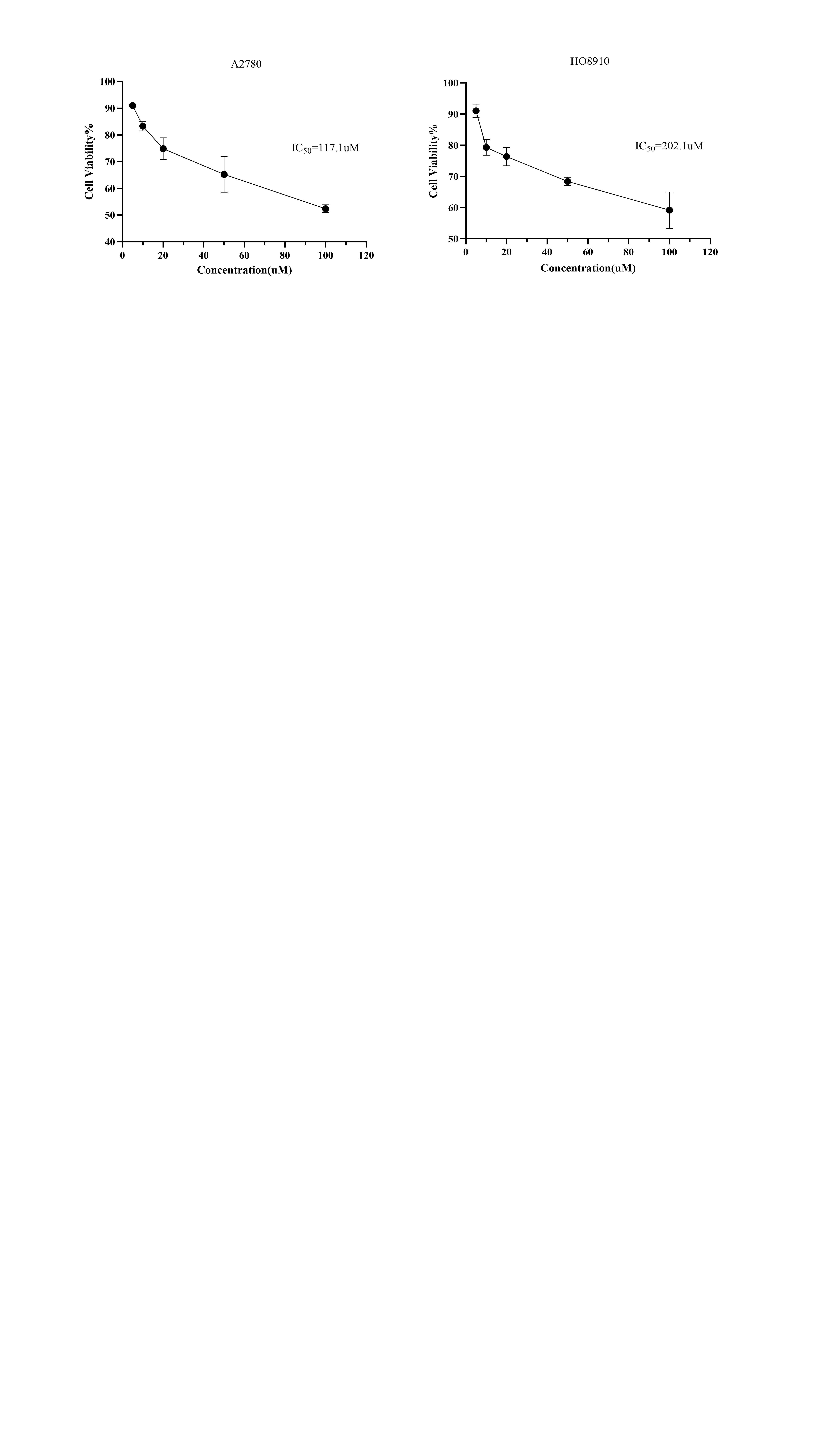

Supplement: Supplementary file 1 [file Image1.TIF]
