# Supplementary material for: Myricetin suppresses TGF-β-induced epithelial-to-mesenchymal transition in ovarian cancer
Source: Front Pharmacol. 2023 Nov 9;14:1288883. doi: 10.3389/fphar.2023.1288883 (PMC10665490; doi:10.3389/fphar.2023.1288883)
Supplement: Supplementary file 2 [file DataSheet1.docx]

**Supplementary Figure Legends**

**Supplementary Figure 1** The IC50 of A2780 and HO8910 by the results from the CCK8 experiment. Data were shown as mean ± SD with three times replication.
